# A comparative study of the effects of genistein and 2-methoxyestradiol on the proteolytic balance and tumour cell proliferation

**DOI:** 10.1038/sj.bjc.6690315

**Published:** 1999-04

**Authors:** I Fajardo, A R Quesada, I Núñez de Castro, F Sánchez-Jiménez, M Á Medina

**Affiliations:** Laboratorio de Bioquímica y Biología Molecular, Facultad de Ciencias, Universidad de Málaga, E-29071 Málaga, Spain

**Keywords:** angiogenesis, urokinase, plasminogen activator inhibitor, matrix metalloproteinase, tissue inhibitor of matrix metalloproteinase

## Abstract

The cytotoxicity of two compounds described as anti-angiogenic, the isoflavone genistein and the oestrogen metabolite 2-methoxyestradiol, has been studied in different human tumour cell lines. Since the degradation of the extracellular matrix is one of the essential steps in angiogenesis, the potential modulatory effects of both compounds on the proteolytic balance in media conditioned by different human tumour cells have been also investigated. The IC_50_ values for 2-methoxyestradiol were lower than those for genistein on all the cell lines tested. In all the cell lines expressing measurable amounts of active enzymes, genistein induced a shift towards antiproteolysis in both matrix metalloproteinase/tissue inhibitor of metalloproteinase and urokinase/plasminogen activator inhibitor proteolytic balances. On the other hand, 2-methoxyestradiol did not produce any clear net shift of the proteolytic balance, with the significant exception of the matrix metalloproteinase/tissue inhibitor of metalloproteinase balance in WAC-2 cells, a neuroblastoma cell line with enhanced expression of the N-*myc* oncogene. © 1999 Cancer Research Campaign


*British Journal of Cancer* (1999) **80**(1/2), 17–24
© 1999 Cancer Research Campaign
Article no. bjoc.1998.0315

### A comparative study of the effects of genistein and 2-methoxyestradiol on the proteolytic balance and tumour cell proliferation

**I Fajardo, AR Quesada, I Núñez de Castro, F Sánchez-Jiménez and MÁ Medina**

Laboratorio de Bioquímica y Biología Molecular, Facultad de Ciencias, Universidad de Málaga, E-29071 Málaga, Spain

**Summary** The cytotoxicity of two compounds described as anti-angiogenic, the isoflavone genistein and the oestrogen metabolite 2-methoxyestradiol, has been studied in different human tumour cell lines. Since the degradation of the extracellular matrix is one of the essential steps in angiogenesis, the potential modulatory effects of both compounds on the proteolytic balance in media conditioned by different human tumour cells have been also investigated. The $IC_{50}$ values for 2-methoxyestradiol were lower than those for genistein on all the cell lines tested. In all the cell lines expressing measurable amounts of active enzymes, genistein induced a shift towards antiproteolysis in both matrix metalloproteinase/tissue inhibitor of metalloproteinase and urokinase/plasminogen activator inhibitor proteolytic balances. On the other hand, 2-methoxyestradiol did not produce any clear net shift of the proteolytic balance, with the significant exception of the matrix metalloproteinase/tissue inhibitor of metalloproteinase balance in WAC-2 cells, a neuroblastoma cell line with enhanced expression of the N-*myc* oncogene.

**Keywords:** angiogenesis; urokinase; plasminogen activator inhibitor; matrix metalloproteinase; tissue inhibitor of matrix metalloproteinase

It is well-established that malignant tumour invasion and metastasis involve a cascade of linked sequential events (Liotta et al, 1983). In addition to the loss of cell growth control, an imbalanced regulation of motility and proteolysis appears to be required for invasion and metastasis. An essential step is the breakdown and removal of extracellular matrix (ECM), mediated by several proteases, the most important of which appear to be matrix metalloproteinases (MMP) and serine proteases (Liotta et al, 1980; Danø et al, 1985; Edmonard and Grimaud, 1990). In fact, the MMPs, 72 kDa and 92 kDa type IV collagenases (MMP-2 and MMP-9 respectively), and the serine proteinase, urokinase-type plasminogen activator (uPA), are mainly involved in tumour invasion and metastasis (Blasi et al, 1986). On the other hand, plasminogen activator inhibitors (PAIs) and tissue inhibitors of metalloproteinases (TIMPs) are naturally occurring protease inhibitors (Andreasen et al, 1990; Liotta et al, 1991). The activity ratios uPA/PAI, MMP9/TIMP1 and MMP2/TIMP2 are maintained at a delicate balance under physiological conditions, but a temporary shift towards proteolysis is needed during tissue remodelling and normal angiogenesis (Montesano, 1992).

An imbalance of positive and negative regulation appears to be required for tumour angiogenesis, invasion and metastasis (Liotta et al, 1991). Pathological angiogenesis induced by solid tumours and required for their progression seems to be the result of an imbalance between angiogenic factors and inhibitors (Folkman and Shing, 1992; Folkman, 1995). It is postulated that a modulation of the protease/inhibitor or angiogenic factor/anti-angiogenic factor ratios leading to a radical decrease of any of them (that is, a shift towards inhibition of proteolytic and/or angiogenic activities) could lead to tumour regression and/or micrometastasis dormancy (Holmgren et al, 1995). Recently, a dietary-derived tyrosine kinase inhibitor and an endogenous oestrogen metabolite, namely, genistein and 2-methoxyestradiol, have been isolated from human urine and they have been shown to inhibit angiogenesis (Schweigerer et al, 1992; Fotsis et al, 1993, 1994). Since breakdown and removal of ECM is a feature shared by both angiogenesis and tumour invasion, it seemed interesting to us to study whether these compounds, genistein and 2-methoxyestradiol, can modulate the proteolytic balances in tumour cells. It was also interesting to study the relationships between their potential modulatory capacity and their ability to inhibit tumour cell growth. The present work summarizes the data we have obtained in a comprehensive comparative study of the effects of genistein and 2-methoxyestradiol on both cell proliferation and proteolytic balance in different human tumour cell lines.

*Received 13 May 1998*
*Revised 4 August 1998*
*Accepted 6 November 1998*

*Correspondence to:* MÁ Medina

### MATERIAL AND METHODS

### Materials

Culture media, serum and antibiotics were from Gibco (Gent, Belgium) and BioWhittaker (USA) and Trasylol from Bayer (Leverkusen, Germany). All other reagents were molecular biology grade and supplied by Sigma (St Louis, MO, USA), Bio-Rad (Hercules, CA, USA), Merck (Darmstad, Germany) and Fluka (Buchs, Switzerland).

### Cell culture

All the cell lines mentioned below were cultured in 10 cm-diameter cell culture plates at 37°C under 5% carbon dioxide, in media containing streptomycin, penicillin and amphoterycin, and supplemented with 10% fetal calf serum (with the exception of WAC2 cells). Among the cells used in the present work, there were human breast cancer (MCF-7, MDA-MB231, ZR-75-1, BT-474, Hs578T and SKBR-3), neuroblastoma (LAN-5 and WAC2), colon adenocarcinoma (HT-29), fibrosarcoma (HT-1080), osteosarcoma (U2-OS) and rhabdomyosarcoma (A-204) cell lines. WAC2 cells are stable transfected neuroblastoma cells derived from the SH-EP line and containing a plasmid with the coding sequence for the N-*myc* oncogene (Schweigerer et al, 1990); these cells were grown in RPMI-1640 medium supplemented with 10% calf serum and selected by the addition of geneticin (200 μg $ml^{-1}$) to the culture medium. BT-474, MDA-MB231, SKBR-3, ZR-75-1 and LAN-5 cells were grown in RPMI-1640 medium; A-204, HT-1080 and Hs578T cells were grown in Dulbecco's modified Eagle medium (DMEM) (with the addition of insulin, 10 μg $ml^{-1}$, in the case of Hs578T cells); MCF-7 and HT-29 cells were grown in DMEM–F12 medium; and U2-OS were grown in McCoy's 5a medium. Cell counts were carried out with a Coulter counter.

### Conditioned media

To prepare conditioned media, cells were grown in 6-well plates. When the cells were subconfluent, medium was aspirated, cells were washed twice with phosphate-buffered saline (PBS) and each well received 1 ml of culture medium without serum and with 200 KIU of Trasylol $ml^{-1}$. Additionally, some wells received genistein (50 μM) or 2-methoxyestradiol (10 μM). After 24 h of incubation, conditioned media were collected and centrifuged at 1000 ***g*** for 20 min. Afterwards, the supernatants were collected and used for zymography. The cells were washed twice with PBS and photographed under phase contrast in a Nikon Diaphot-TMD microscope. Duplicates were used to determine cell number.

### Assays of toxicity

To determine the $IC_{50}$ values of the agents tested, the 3-(4,5-dimethylthiazol-2-yl)-2,5-diphenyltetrazolium bromide (MTT) dye reduction assay was carried out as previously described (Mossmann, 1983).

### Zymographies for PA and PAI activities

Aliquots of conditioned media normalized for equal cell numbers were subjected to sodium dodecyl sulphate-polyacrylamide gel electrophoresis (SDS-PAGE) at 4°C under non-reducing conditions, with 3% stacking gel and 10% resolving gel. Gels were washed for 10 min twice with 2.5% Triton X-100 and twice with PBS and layed over a substrate gel prepared with agar (0.8%), plasminogen (40 μg $ml^{-1}$) and skimmed milk (1.5% in PBS). Gels were incubated under a moist atmosphere overnight at 4°C and afterwards they were incubated at 37°C. After 4–8 h, bands of proteolysis due to uPA activity were photographed under dark field. After an additional day of incubation at room temperature, the only white areas under dark field illumination (due to the presence of skimmed milk protein) were those corresponding to bands protected against proteolytic activity, and they represent the bands of PAI activity.

### Direct and reverse gelatinolytic assays

The gelatinolytic activity of MMP-2 and MMP-9 delivered to the conditioned media by the different tumour cells was detected in gelatinograms as follows. Samples were subjected to non-reducing SDS-PAGE as above but with gelatin (1 mg $ml^{-1}$) added to the 10% resolving gel. After electrophoresis, gels were washed twice with 50 mM Tris-HCl, pH 7.4, supplemented with 2% Triton X-100, and twice with 50 mM Tris-HCl, pH 7.4. Each wash was for 10 min and with continuous shaking. After the washes, the gels were incubated at 37°C for 24 h immersed in a substrate buffer (50 mM Tris-HCl, pH 7.4, supplemented with 1% Triton X-100, 5 mM calcium chloride and 0.02% $Na_3N$). Afterwards, the gels were stained with Commassie blue R-250 and the bands of gelatinase activity could be detected as non-stained bands in a dark, stained background. To detect stromelysin, caseinograms were carried out as described for gelatinograms, using α- or β-casein at 1 mg $ml^{-1}$ instead of gelatin.

To detect TIMP activities some modifications were required. Media conditioned by PMA-treated HT-1080 cells were treated with 2% SDS at room temperature for 30 min and added (20% v/v) to the 12% resolving gel. Prior to the addition of the stacking gel, an electrophoretic run was carried out until the moment in which the front line containing the phenol red of the conditioned medium reached the end of the gel. From here on the procedure was the same as in the case of direct gelatinolytic assay. In this case, the bands of TIMP activity could be detected as dark bands over a clearer background.

### Quantitative analysis of bands of activity

Bands of activity in the zymograms and gelatinograms were quantified with an lbas image analyser.

### RESULTS

### Effects of genistein and 2-methoxyestradiol on tumour cell proliferation

The effects of both compounds, genistein and 2-methoxyestradiol, have been tested on a wide range of human tumour cell lines, including breast cancer (BT-474, Hs578T, MCF-7, MDA-MB231, ZR-75-1), colon adenocarcinoma (HT-29), neuroblastoma (LAN-5, WAC2), osteosarcoma (U2-OS), rhabdomyosarcoma (A-204) and fibrosarcoma (HT-1080) cell lines.

First of all, it was necessary to access the growth rate curves obtained for the different cell lines in the culture conditions of our lab. As expected, the profiles of all the curves were similar, though the proliferation rates varied in a wide range (Figure 1). Among breast cancer cell lines, the most proliferant one was MDA-MB231, a hormone-insensitive cell line. MCF-7 cells had a medium proliferation rate, and all the other breast cancer cell lines showed the lowest proliferation rates. Both rhabdomyosarcoma A-204 and fibrosarcoma HT-1080 cells showed complete growth curves in the period tested with regression of the populations from the fourth day; their proliferation rates were medium. Osteosarcoma U2-OS cells also had a medium proliferation rate but they grew continuously from the second to the sixth day showing no sign of regression. Finally, the highest proliferation rates were those of colon adenocarcinoma HT-29 and neuroblastoma (WAC2, LAN-5) cell lines, with decay of the growth from the fourth day on, only for LAN-5 cells.

© Cancer Research Campaign 1999

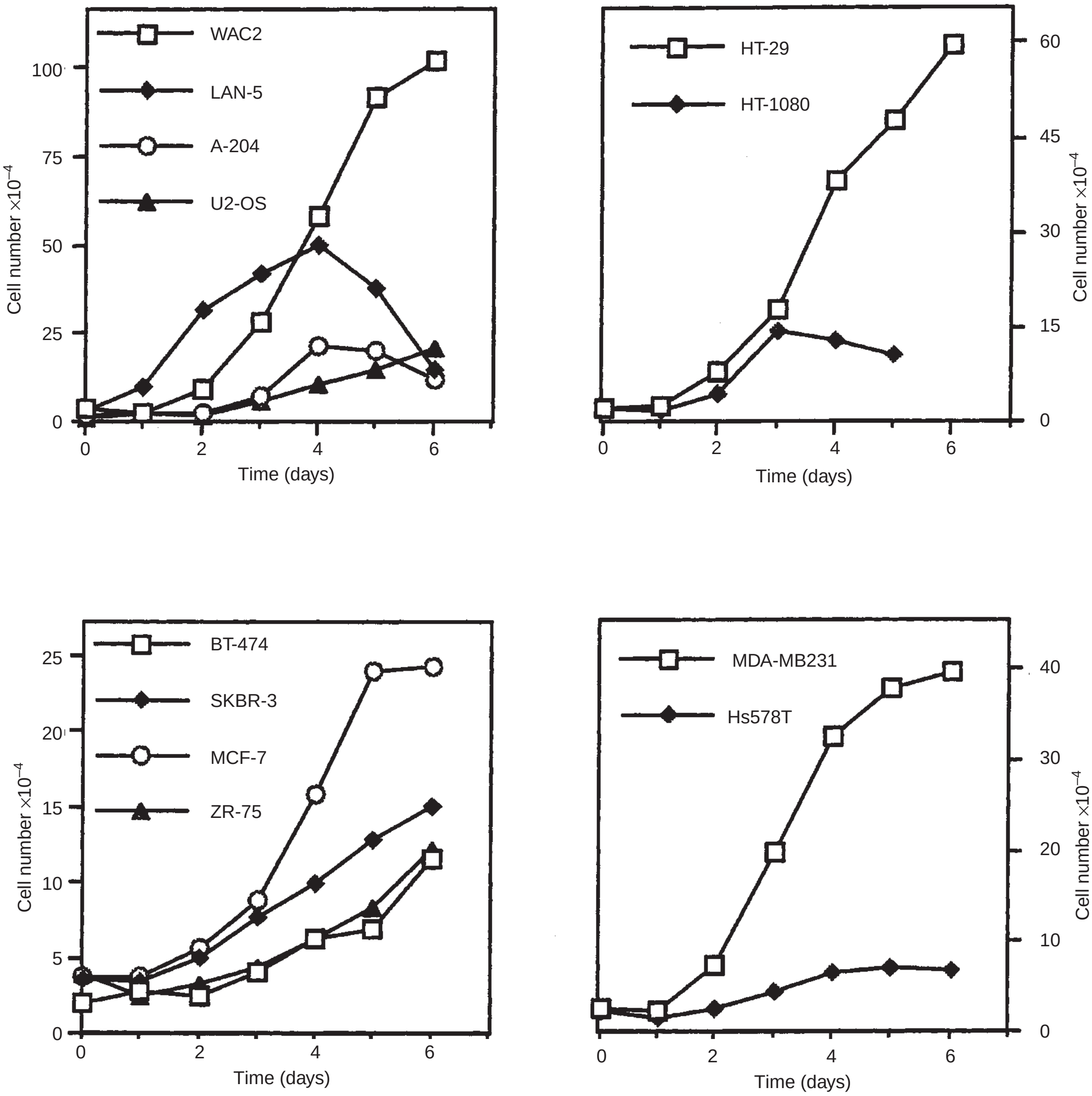

**Figure 1** Growth profiles for the different human tumour cell lines tested under the culture conditions described in Material and Methods

The $IC_{50}$ values for both compounds were determined by functional viability assays with MTT (Table 1). There were no clear correlations among $IC_{50}$ values and the proliferation rates or the cell types. Significantly, among breast cancer cell lines, the hormone-independent MDA-MB231 cell line was much more sensitive to toxicity by both compounds than the other, hormone-dependent breast cancer cell lines tested. In all the cases, $IC_{50}$ values for 2-methoxyestradiol were much lower than those for genistein.

Figures 2 and 3 show the typical morphological changes induced by both treatments on some of the cell lines tested. Genistein-treated cells emitted projections, suggesting that they had acquired a differentiated phenotype. Some cells resembled apoptotic cells with blebbing of the cytoplasm. On the other hand, 2-methoxyestradiol-treated cells losed their shape and acquired a more rounded aspect.

### Screening of proteases and their inhibitors in media conditioned by tumour cells

Table 2 summarizes the results of the screening of the ECM proteases and protease inhibitors delivered by the tumour cells to the culture media carried out in the present work. None of the activities which were tested could be detected in media conditioned by the low proliferation rate BT-474 and ZR-75-1 breast cancer cells. We also failed to detect any of the activities in the medium conditioned by the highly proliferant LAN-5 neuroblastoma cells. Therefore, these three cell lines could not be further used in the study of the potential modulatory effects of genistein and 2-methoxyestradiol on the proteolytic balances.

The most interesting results were obtained with the media conditioned by A-204, Hs578T, HT-1080, MDA-MB231, U2-OS and WAC2 cells, which were further analysed. Only one of the

© Cancer Research Campaign 1999

**Table 1** Comparison of the antiproliferative effect of genistein and 2-methoxyestradiol on human tumor cell lines

| | $IC_{50}$ (μM) | |
|---|---|---|
| **Tumour line** | **Genistein** | **2-Methoxyestradiol** |
| BT-474 | 33 | 12.0 |
| Hs578T | 65 | 1.0 |
| MCF-7 | 72 | 3.0 |
| MDA-MB231 | 23 | 0.4 |
| SKBR-3 | 65 | 2.7 |
| ZR-75 | 128 | 31.0 |
| A-204 | 25 | 0.7 |
| HT-1080 | 100 | 1.6 |
| HT-29 | 32 | 5.5 |
| LAN-5 | 25 | 1.0 |
| U-2OS | 185 | 2.3 |
| WAC2 | 24 | 0.8 |

**Table 2** Screening for the detection of ECM proteases and protease inhibitors by zymograms[a]

| Tumour line | MMP-9 | MMP-2 | Stromelysins | TIMPs | uPA | PAIs |
|---|---|---|---|---|---|---|
| BT474 | – | – | – | – | – | – |
| Hs578T | – | + | – | + | – | – |
| MCF-7 | – | – | – | + | – | – |
| MDA-MB231 | – | – | – | + | +[b] | +[c] |
| SKBR-3 | – | + | – | – | – | – |
| ZR-75 | – | – | – | – | – | – |
| A-204 | – | – | – | + | + | – |
| HT-1080 | + | + | – | + | + | – |
| HT-29 | – | – | – | + | – | – |
| LAN-5 | – | – | – | – | – | – |
| WAC2 | – | + | – | + | + | + |
| U2-OS | + | + | – | + | – | + |

[a]The activities detected are indicated by + and those undetectable under the experimental conditions are indicated by –. [b]uPA activity could only be detected in media conditioned by genistein-treated cells. [c]PAI activity could only be detected in media conditioned by 2-methoxyestradiol-treated cells.

activities tested was detected in the media conditioned by HT-29 and MCF-7 (TIMP activity) and SKBR-3 cells (MMP-2 activity).

Since MMP-9 activity was only detected in HT-1080 and U2-OS cells, we restricted our study to the evaluation of the MMP-2/TIMP-2 and uPA/PAI proteolytic balances.

### Effects of genistein and 2-methoxyestradiol on type IV collagenase and TIMP activities

Figure 4 summarizes the results obtained with gelatinolytic assays in both direct and reverse zymograms. As mentioned above, 92 kDa-type IV collagenase (MMP-9) was only detected in the media conditioned by HT-1080 and U2-OS cells. In reverse zymograms, two bands of TIMP activity were detected but we only further analysed the most prominent one, that corresponding to TIMP-2. Table 3 shows the normalized quantitative data obtained by digital analysis of the gelatinograms. In all the cases tested, genistein induced a significant decrease in gelatinolytic activities (greater for MMP-2 activity than for MMP-9 activity). Furthermore, in HT-1080 and WAC-2 cells a parallel increase in the size of TIMP-2 activity bands was observed. Both effects accounted for an important decrease in the MMP-2/TIMP-2 proteolytic balance.

The results obtained with 2-methoxyestradiol were less conclusive. In fact, in media conditioned by Hs578T-, HT-1080- or U2-OS-treated cells, there were simultaneous increases in both gelatinolytic and TIMP activities with no significant changes in the proteolytic balance. On the contrary, in media conditioned by WAC2-treated cells, there were more pronounced increases in the TIMP activities than those produced in the gelatinolytic activities,

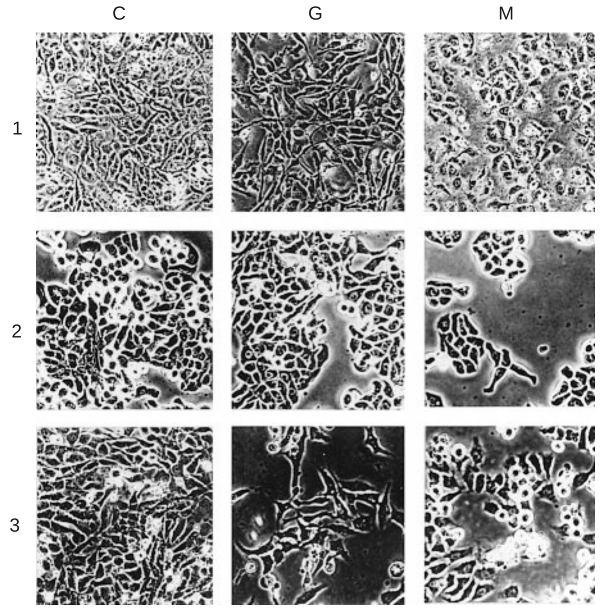

**Figure 2** Effects of genistein (G) and 2-methoxyestradiol (M) on the morphology of cultured human tumour HT-1080 (1), HT-29 (2) and WAC2 (3) cells, as compared to control cells (C)

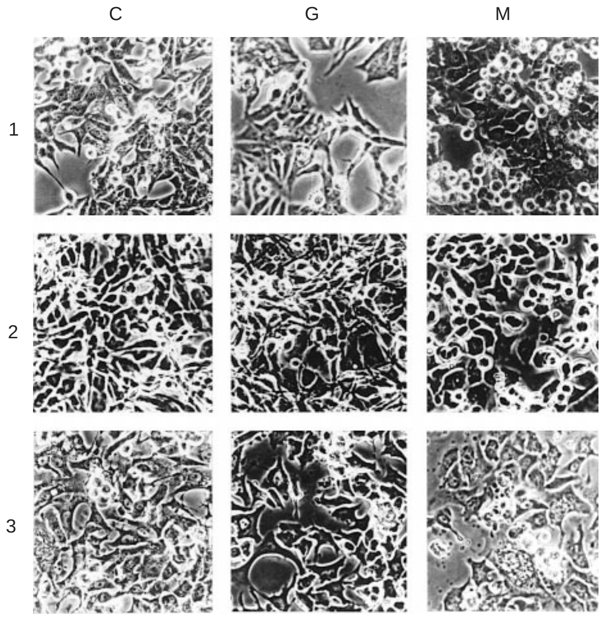

**Figure 3** Effects of genistein (G) and 2-methoxyestradiol (M) on the morphology of cultured human tumour MCF-7(1), MDA-MB231 (2) and SKBR-3 (3) cells, as compared to control cells (C)

 *© Cancer Research Campaign 1999*

**Table 3** Quantification of MMP-2 and TIMP-2 activities by image analysis

| Treatment/cell line | MMP-2 activity[a] | TIMP-2 activity[a] | MMP-2/TIMP-2[b] |
|---|---|---|---|
| Genistein | | | |
| Hs578T | 0.82 ± 0.07[c] | 1.08 ± 0.20 | 0.78 ± 0.11[c] |
| HT-1080 | 0.72 ± 0.02[c] | 1.20 ± 0.02[c] | 0.60 ± 0.01[c] |
| U2-OS | 0.75 ± 0.12[d] | 0.99 ± 0.16 | 0.75 ± 0.08[c] |
| WAC2 | 0.77 ± 0.01[c] | 1.37 ± 0.10[c] | 0.54 ± 0.00[c] |
| 2-Methoxyestradiol | | | |
| Hs578T | 1.10 ± 0.10 | 1.16 ± 0.25 | 0.97 ± 0.14 |
| HT-1080 | 0.88 ± 0.25 | 1.26 ± 0.24 | 0.71 ± 0.24[d] |
| U2-OS | 1.21 ± 0.28 | 1.17 ± 0.17 | 1.08 ± 0.36 |
| WAC2 | 1.09 ± 0.03[d] | 1.36 ± 0.04[c] | 0.79 ± 0.01[c] |

[a]Results are means ± s.d. of three different experiments, except in the case of WAC2 cells, where they are means of two different experiments. Results are given as the ratios of activities (treated cells/control cells). Thus, a value below 1 indicates a decrease and a value over 1 indicates an increase in activity as compared to control values. [b]These are ratios of the values given in the two previous columns. Here, a value below 1 indicates that the treatment induced a shift towards antiproteolysis and a value over 1 indicates that the treatment induced a shift towards an increased proteolysis. [c]Significant versus control values ($P$<0.01) and [d]significant versus control values ($P$<0.05), according to a non-parametric Mann–Whitney's $U$-test.

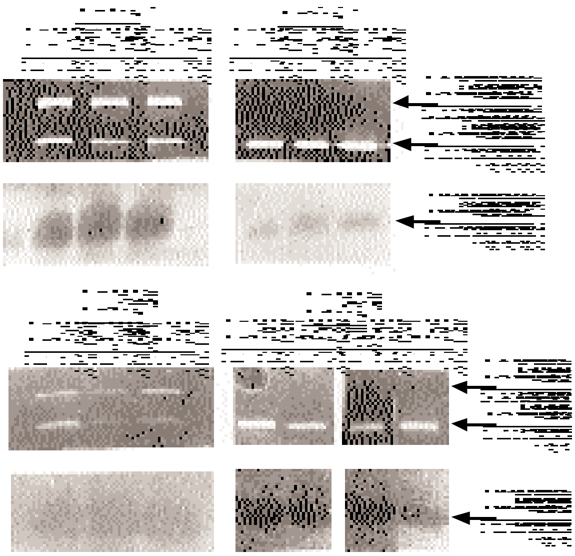

**Figure 4** Bands of gelatinolytic activities (MMP-9 and MMP-2) or TIMP-2 activity detected by direct or reverse gelatinograms, respectively, of media conditioned by human tumour HT-1080 (**A**), Hs578T (**B**), U2-OS (**C**), or WAC-2 (**D**). Treatments: control, untreated cells (C), 50 μM genistein (G), or 10 μM 2-methoxyestradiol (M)

**Table 4** Quantification of plasminogen activator and plasminogen activator inhibitor activities by image analysis[a]

| Treatment/tumour line | uPA activity | PAI activity |
|---|---|---|
| Genistein | | |
| A-204 | 0.30 | – |
| HT-1080 | 0.52 | – |
| U2-OS | – | 1.30 |
| WAC2 | 0.00 | 12.00 |
| 2-Methoxyestradiol | | |
| A-204 | 1.65 | – |
| HT-1080 | 1.26 | – |
| U2-OS | – | 0.94 |
| WAC2 | 2.14 | – |

[a]Quantitative data here shown are the results of image analysis of data shown in Figure 2. They are typical results and they are given as the ratios of activities (treated cells/control cells). Thus, a value below 1 indicates a decrease and a value over 1 indicates an increase in activity as compared to control values. A minus sign indicates that there was no detectable activity in control cells under the experimental conditions.

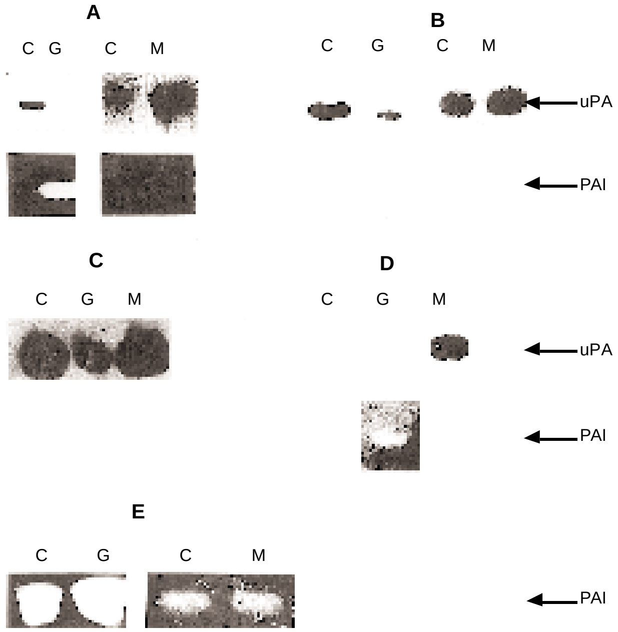

**Figure 5** Bands of uPA or PAI activities detected by plasminogen zymography of media conditioned by human tumour WAC2 (**A**), A-204 (**B**), HT-1080 (**C**), MDA-MB231 (**D**) or U2-OS (**E**). Treatments: control, untreated cells (C), 50 μM genistein (G), or 10 μM 2-methoxyestradiol (M).

giving rise to a significant decrease of the proteolytic balance (Table 3). In media conditioned by 2-methoxyestradiol-treated SKBR-3 cells, the only activity detected, namely that corresponding to MMP-2, was apparently increased by 25% as compared to control, untreated cells (results not shown).

### Effects of genistein and 2-methoxyestradiol on uPA and PAI activities

Figure 5 shows typical results obtained in the zymograms carried out to detect PA and PAI activities, and Table 4 shows the normalized quantitative data obtained by digital analysis of the zymograms. As it was the case for the MMP-2/TIMP-2 balance, genistein strongly modified the uPA/PAI balance to antiproteolysis in WAC2 cell conditioned media by simultaneous increase of PAI and decrease of uPA activities. It is noteworthy to remark the dramatic increase in PAI activity detected in the medium conditioned by genistein-treated WAC2 cells as compared to untreated cells. In all the cases, genistein produced a pronounced decrease in uPA and an increase in PAI activity.

On the other hand, 2-methoxyestradiol increased uPA activity in A-204, HT-1080 and WAC2 cell conditioned media and it produced no significant effect on the PAI activity detected in U2-OS conditioned media.

© Cancer Research Campaign 1999 

The case of MDA-MB231 cells is interesting: media conditioned by control, untreated cells contained no detectable uPA or PAI activities; however, the treatment with 50 μM genistein gave rise to a detectable band of PAI activity and, on the contrary, the treatment with 10 μM 2-methoxyestradiol gave rise to a detectable band of uPA activity.

### DISCUSSION

### Modulation of tumour cell proliferation

The interest in genistein as a potential anti-cancer agent can be traced back to the seminal work by Akiyama *et al* (1987) which firstly showed that it was a specific and quite potent inhibitor of protein tyrosine phosphorylation. It has been shown that genistein inhibits both oestrogen and growth factor stimulated proliferation of human breast cancer cells (Peterson and Barnes, 1996). This dual role of genistein is consistent with its previously proposed role as agonist/antagonist of oestrogen activity (Barnes et al, 1994). In fact, genistein inhibits the growth of both oestrogen-dependent and oestrogen-independent breast cancer cells, as firstly reported by Peterson and Barnes (1991). The interest in genistein as a potential therapeutic agent was reinforced when it was isolated from human urine and it was shown to inhibit tumour cell growth in some paediatric tumour cell lines and in some models of neoplasia in vivo (Schweigerer et al, 1992; Fotsis et al, 1993). Our results show that both compounds are inhibitory for the growth of a wide range of tumour cells. In particular, our data confirm that previously reported by Peterson and Barnes (1991), stating that genistein can inhibit the proliferation of human breast cancer cells. At the same time, the cytotoxicity of these compounds was much lower on non-neoplastic cells (results not shown). The morphological changes induced by genistein in highly proliferant tumour cells suggest that this compound could contribute to a reversion of the tumoural phenotype. Interestingly, it has been shown that genistein can induce some cancer cells to enter apoptosis (Gorczyca et al, 1993).

As was the case for genistein, 2-methoxyestradiol was also isolated from human urine and it was shown to inhibit tumour cell growth in some paediatric tumour cell lines and in some models of neoplasia in vivo (Fotsis et al, 1994). We have found that, in general, 2-methoxyestradiol was more toxic to tumour cells than genistein. On the other hand, the effect induced by 2-methoxyestradiol on cell morphology reinforces the mechanism of action suggested by Fotsis et al (1994): as 2-methoxyestradiol-treated cells lose their shape, it could be speculated that this is the result of some kind of cytoskeleton disruption. In fact, it has been shown that 2-methoxyestradiol acts as a ligand for tubulin by binding to the colchicin site of this cytoskeleton protein, inhibiting the assembly of the tubulin monomer or the stability of the tubulin polymer (D'Amato et al, 1994). This interference with the microtubular net could explain the potent anti-tumour effect of 2-methoxyestradiol. Due to the similarity in the actions of both agents, taxol and 2-methoxyestradiol, it could be suggested that 2-methoxyestradiol could induce apoptosis, as taxol does, and that this induction occurs in parallel to an activation of $p34^{cdc2}$ (Donaldson et al, 1994); this suggestion remains to be tested.

### Modulation of the proteolytic balance

A second common point of interest of genistein and 2-methoxyestradiol is their demonstrated capacity to inhibit angiogenesis (Fotsis et al, 1993, 1994), because of the fact that solid tumour progression and metastasis are dependent on angiogenesis (Folkman and Shing, 1992). An essential step in cancer invasion, angiogenesis and metastasis is a proteolytic degradation of ECM (Liotta et al, 1991). A positive correlation among tumour invasiveness and ECM protease levels has been shown (Kohn and Liotta, 1995). On the other hand, experimental evidences accumulate for a negative correlation between TIMP activities and the metastasic potential of tumour cells (Álvarez et al, 1990; Tsuchiya et al, 1993). Thus, it seems that there is a direct relationship between an imbalance of the proteolytic equilibrium towards proteolysis and the onset of tumour invasion and metastasis. In fact, Packman et al (1995) have found in thyroid follicular carcinoma that both uPA and type IV-collagenases are expressed at higher levels in a more invasive cell line (FTG-238) than in a less invasive one (FTG-133). Lau et al (1995) have found in colorectal adenocarcinoma samples higher level of uPA and its receptor than in their corresponding healthy, control samples. Stearns et al (1993) have found a positive correlation among MMP2 expression and malignancy in bladder cancer.

Two main conclusions can be derived from all these findings. First, the evaluation of the expression levels of ECM proteases and their inhibitors could have a prognostic value. In fact, it has been shown that both MMP-9/TIMP-1 and MMP-2/TIMP-2 ratios are lower in cervix carcinoma with a good prognosis than in those with a bad prognosis (Nuovo et al, 1995) and that the MMP-2/TIMP-2 ratio is high in relapsed urothelial cancers (Gohji et al, 1996). It should be stressed that the changes in the values of the protease/inhibitor ratios are more significant than the changes only in the levels of either proteases or inhibitors. Our own results shown here support this hypothesis and seem to indicate that zymograms are convenient, easy, rapid, sensitive and economic procedures to evaluate the proteolytic balance. However, the use of the proteolytic balance values as prognostic indicators requires the establishment of acceptable reference patterns as well as the determination of the cancer types where this method could be useful.

A second hypothesis is that the modulation of the proteolytic balance towards antiproteolysis could avoid tumour invasion and metastasis. In fact, this suggestion is also supported by several experimental data (Liotta et al, 1991; Montesano, 1992). Thus, the modulation of the proteolytic balance could be a promising anti-tumour strategy, and it would be noteworthy to promote the search for new compounds with a potential capacity to shift the proteolytic balance towards antiproteolysis.

Genistein has been described as a natural inhibitor of tumour cell growth (Peterson and Barnes, 1991; Schweigerer et al, 1992). Afterwards, it has been shown that genistein is an inhibitor of angiogenesis; in fact, genistein inhibits vascular endothelial cell proliferation through collagenic matrices as newly formed capilars (Fotsis et al, 1993). It was postulated that the anti-angiogenic effect of genistein could be due to its effect on ECM proteases and their inhibitors. This hypothesis was initially supported by the reported inhibitory effect of genistein on both uPA and PAI activities delivered to culture media conditioned by vascular endothelial cells (Fotsis et al, 1993). Recently, we have shown that genistein reduces the levels of MMP-2 activity in some paediatric tumour cell lines (García de Veas et al, 1995). The present report expands our previous preliminary observations to a wider range of tumours, and emphasizes the modulatory effect of genistein on the MMP-2/TIMP-2 and uPA/PAI ratios towards antiproteolysis in different

© Cancer Research Campaign 1999

tumour cell lines; interestingly, this antiproteolytic effect is the result of both inhibition of proteases and activation of protease inhibitors.

On the other hand, since 2-methoxyestradiol has been described as an anti-angiogenic compound able to inhibit the induction of uPA activity by basic fibroblast growth factor in vascular endothelial cells (Fotsis et al, 1994), we postulated that this compound could modulate the proteolytic balance in a similar way to that proven for genistein. This was not the case, as demonstrated in the present work. Our results show that 24 h of treatment with 10 μM 2-methoxyestradiol induces either an apparent increase of uPA activity or an apparent decrease of PAI activity. The effects of 2-methoxyestradiol on the MMP-2/TIMP-2 ratio seem to be dependent on the cell line tested. Thus, it seems that the anti-angiogenic effect of 2-methoxyestradiol is not only due to a modulation of the proteolytic balance. Taking into account the effects of this compound on cell morphology caused by its capacity to bind cytoskeleton proteins, we suggest that its anti-angiogenic effect could be produced by some interferences in the first step of adhesion to the ECM previous to its degradation; interferences in the posterior step of migration cannot be ruled out.

### CONCLUSION

It seems that this new strategy of searching for efficient and selective modulators of the proteolytic balance could be very promising for cancer research; furthermore, these findings would be relevant for the design and establishment of new therapeutic strategies against other diseases in which a pathological angiogenesis is involved. However, it should be kept in mind that tumour promotion, invasion and metastasis are very complex phenomena in which many gene products other than ECM proteases and their inhibitors are involved. In the present comparative study, we demonstrate that genistein is an anti-angiogenic compound able to modulate the proteolytic balance towards antiproteolysis, but the anti-angiogenic compound 2-methoxyestradiol has no clear antiproteolytic effect. Other possibilities remain to be elucidated in further experimental efforts.

In addition to the potential therapeutic value of genistein, a dietary-derived tumour cell growth inhibitor, its preventive value should be stressed. In fact, people in some Asian contries consume on the average 20–50 times more soy products (rich in genistein) than in Western countries (Messina et al, 1994), where breast, prostate and bladder cancer mortalities are higher (Dunn, 1975; Severson et al, 1989; Parker et al, 1996).

### ACKNOWLEDGEMENTS

This work was partially supported by a Grant of the University of Málaga (MAM), DGICYT Grant SAF98-0150 (FSJ) and funds from PAI #3309 from the Junta de Andalucía (INC).

### REFERENCES

Akiyama T, Ishida I, Nakagawa S, Ogawara H, Watanabe S, Itoh N, Shibuya M and Fuami Y (1987) Genistein, a specific inhibitor of tyrosine-specific protein kinases. *J Biol Chem* **262**: 5592–5595

Alvarez O, Carmichael DF and Declerk YA (1990) Inhibition of collagenolytic activity and metastasis of tumor cells by a recombinant human tissue inhibitor of metalloproteinases. *J Natl Cancer Inst* **82**: 589–595

Andreasen PA, Georg B, Lund LR, Riccio A and Stacey SN (1990) Plasminogen activators inhibitors: hormonally regulated serpins. *Mol Cell Endocrinol* **68**: 1–19

Barnes S, Peterson G, Grubbs C and Setchell K (1994) Potential role of dietary isoflavones in the prevention of cancer. In *Diet and Cancer*: *Markers, Prevention, and Treatment*, Jacubs MM (ed) pp. 135–147. Plenum Press: New York

Blasi F, Riccio A and Sebatio G (1986) Human plasminogen activators: genes and protein structure. In *Human Genes and Diseases*, Blasi F (ed), pp. 377–414. Wiley: London

D'Amato RJ, Lin CM, Flynn E, Folkman J and Hamel E (1994) 2-Methoxyestradiol, an endogenous mammalian metabolite, inhibits tubulin polymerization by interacting at the colchicine site. *Proc Natl Acad Sci USA* **91**: 3964–3968

Danø K, Andreasen PA, Grøhndahl-Hansen J, Kristensen P, Nielsen LS and Skriver L (1985) Plasminogen activators, tissue degradation and cancer. *Adv Cancer Res* **44**: 139–266

Donaldson KL, Goolsby G, Kiener PA and Wahl AF (1994) Activation of $p34^{dcd2}$ coincident with taxol-induced apoptosis. *Cell Growth Differ* **5**: 1041–1050

Dunn JE (1975) Cancer epidemiology in populations of the United States – with emphasis on Hawaii and California – and Japan. *Cancer Res* **35**: 3240–3245

Edmonard H and Grimaud JA (1990) Matrix metalloproteinases. A review. *Cell Mol Biol* **36**: 131–153

Folkman J (1995) Angiogenesis in cancer, vascular, rheumatoid and other disease. *Nature Med* **1**: 27–31

Folkman J and Shing Y (1992) Angiogenesis. *J Biol Chem* **267**: 10931–10934

Fotsis T, Pepper M, Adlercreutz H, Fleischman G, Hase T, Montesano R and Schweigerer L (1993) Genistein, a dietary-derived inhibitor of *in vitro* angiogenesis. *Proc Natl Acad Sci USA* **90**: 2690–2694

Fotsis T, Zhang Y, Pepper MS, Adlercreutz H, Montesano R, Nauroth PP and Schweigerer L (1994) The endogenous oestrogen metabolite 2-methoxyoestradiol inhibits angiogenesis and suppresses tumour growth. *Nature* **368**: 237–239

García de Veas R, Schweigerer L and Medina MA (1995) Matrix metalloproteinase-2 and tissue inhibitor of metalloproteinase-2 expression in paediatric tumor cells. Effects of tumor cell proliferation modulators on gelatinolytic activity. *J Cancer Res Clin Oncol* **121**: 275–278

Gohji K, Fujimoto N, Fujii A, Komiyama T, Okawa J and Nakajima M (1996) Prognostic significance of circulating matrix metalloproteinase-2 to tissue inhibitor of metalloproteinases-2 ratio in recurrence of urothelial cancer after complete resection. *Cancer Res* **56**: 3196–3198

Gorczyca W, Gong J, Ardelt B, Traganos F and Darzynkiewicz Z (1993) The cell cycle related differences in susceptibility of HL-60 cells to apoptosis induced by various antitumor agents. *Cancer Res* **53**: 3186–3192

Holmgren L, O'Reilly MS and Folkman J (1995) Dormancy of micrometastases: balanced proliferation and apoptosis in the presence of angiogenesis suppression. *Nature Med* **1**: 149–153

Kohn EC and Liotta LA (1995) Molecular insights into cancer invasion: strategies for prevention and intervention. *Cancer Res* **55**: 1856–1862

Lau HKF, Kim M, Koo J, Chiu B and Murray D (1995) Increase of a urokinase receptor-related low-molecular-weight molecule in colorectal adenocarcinomas. *Clin Exp Metastasis* **13**: 492–498

Liotta LA, Tryggvason K, Garbisa S, Hart IR, Foltz CM and Shaflie S (1980) Metastatic potential correlates with enzymatic degradation of basement matrix collagen. *Nature* **284**: 67–69

Liotta L, Rao CN and Bardsky SH (1983) Tumor invasion and the extracellular matrix. *Lab Invest* **49**: 639–649

Liotta LA, Steeg PS and Stetler-Stevenson WG (1991) Cancer metastasis and angiogenesis: an imbalance of positive and negative regulation. *Cell* **64**: 327–336

Messina MJ, Persky V, Setchell KDR and Barnes S (1994) Soy intake and cancer risk: a review of the *in vitro* and *in vivo* data. *Nutr Cancer* **21**: 113–131

Montesano R (1992) Regulation of angiogenesis *in vitro*. *Eur J Clin Invest* **22**: 504–515

Mossmann T (1983) Rapid colorimetric assay for cellular growth and survival: application to proliferation and cytotoxicity assays. *J Immunol Method* **65**: 55–63

Nuovo GJ, MacConnell PB, Simsir A, Valea F and French DL (1995) Correlation of the *in situ* detection of polymerase chain reaction-amplified metalloproteinase complementary DNAs and their inhibitors with prognosis in cervical carcinoma. *Cancer Res* **55**: 267–275

Packman KS, Demeure MD, Doffek KM and Wilson SD (1995) Increased plasminogen activator and type IV collagenase activity in invasive follicular thyroid carcinoma cells. *Surgery* **118**: 1011–1017

Parker SL, Tong T, Bolden S and Wingo PA (1996) Cancer statistics. *CA Cancer J Clin* **65**: 5–27

© Cancer Research Campaign 1999

Peterson G and Barnes S (1991) Genistein inhibition of the growth of human breast cancer cells: independence from estrogen receptors and the multi-drug resistance gene. *Biochem. Biophys Res Commun* **179**: 661–667

Peterson G and Barnes S (1996) Genistein inhibits both estrogen and growth factor-stimulated proliferation of human breast cancer cells. *Cell Growth Differ* **7**: 1345–1351

Schweigerer L, Breit S, Wenzel A, Tsunamoto K, Ludwig R and Schwab M (1990) Augmented N-MYC expression advances the malignant phenotype of human neuroblastoma cells: evidence for induction of autocrine growth factor activity. *Cancer Res* **50**: 4411–4416

Schweigerer L, Christeleit K, Fleischmann G, Adlercreutz H, Wahala K, Hase T, Schwab M, Ludwig R and Fotsis T (1992) Identification in human urine of a natural growth inhibitor for cells derived from solid paediatric tumors. *Eur J Clin Invest* **22**: 260–264

Severson RK, Nomura AMY, Grove J-S and Stemmerman GN (1989) A prospective study of demographics, diet, and prostate cancer among men of Japanese ancestry in Hawaii. *Cancer Res* **49**: 1857–1860

Stearns ME and Wang M (1993) Type IV collagenase ($MW_r$ 72,000) expression in human prostate: benign and malignant tissue. *Cancer Res* **53**: 878–883

Tsuchiya Y, Sato H, Endo Y, Okada Y, Mai M, Takuma S and Seiki M (1993) Tissue inhibitor of metalloproteinase 1 is a negative regulator of the metastatic ability of a human gastric cancer cell line, kkls, in the chick embryo. *Cancer Res* **53**: 1397–1402

 *© Cancer Research Campaign 1999*
